# Metastasis-suppressing *NID2*, an epigenetically-silenced gene, in the pathogenesis of nasopharyngeal carcinoma and esophageal squamous cell carcinoma

**DOI:** 10.18632/oncotarget.12889

**Published:** 2016-10-25

**Authors:** Annie Wai Yeeng Chai, Arthur Kwok Leung Cheung, Wei Dai, Josephine Mun Yee Ko, Joseph Chok Yan Ip, Kwok Wah Chan, Dora Lai-Wan Kwong, Wai Tong Ng, Anne Wing Mui Lee, Roger Kai Cheong Ngan, Chun Chung Yau, Stewart Yuk Tung, Victor Ho Fun Lee, Alfred King-Yin Lam, Suja Pillai, Simon Law, Maria Li Lung

**Affiliations:** ^1^ Department of Clinical Oncology, The University of Hong Kong, Hong Kong (SAR), People's Republic of China; ^2^ Center for Cancer Research, The University of Hong Kong, Hong Kong (SAR), People's Republic of China; ^3^ Department of Pathology, The University of Hong Kong, Hong Kong (SAR), People's Republic of China; ^4^ Center for Nasopharyngeal Carcinoma Research, The University of Hong Kong, Hong Kong (SAR), People's Republic of China; ^5^ Department of Clinical Oncology, Pamela Youde Nethersole Eastern Hospital, Hong Kong (SAR), People's Republic of China; ^6^ Department of Clinical Oncology, Queen Elizabeth Hospital, Hong Kong (SAR), People's Republic of China; ^7^ Department of Oncology, Princess Margaret Hospital, Hong Kong (SAR), People's Republic of China; ^8^ Department of Clinical Oncology, Tuen Mun Hospital, Hong Kong (SAR), People's Republic of China; ^9^ Department of Cancer Molecular Pathology, Griffith Medical School and Menzies Health Institute Queensland, Griffith University, Gold Coast, Australia; ^10^ Department of Surgery, The University of Hong Kong, Hong Kong (SAR), People's Republic of China

**Keywords:** Nidogen-2 (NID2), promoter hypermethylation, metastasis, nasopharyngeal carcinoma (NPC), esophageal squamous cell carcinoma (ESCC)

## Abstract

Nidogen-2 (NID2) is a key component of the basement membrane that stabilizes the extracellular matrix (ECM) network. The aim of the study is to analyze the functional roles of NID2 in the pathogenesis of nasopharyngeal carcinoma (NPC) and esophageal squamous cell carcinoma (ESCC). We performed genome-wide methylation profiling of NPC and ESCC and validated our findings using the methylation-sensitive high-resolution melting (MS-HRM) assay. Results showed that promoter methylation of *NID2* was significantly higher in NPC and ESCC samples than in their adjacent non-cancer counterparts. Consistently, down-regulation of *NID2* was observed in the clinical samples and cell lines of both NPC and ESCC. Re-expression of NID2 suppresses clonogenic survival and migration abilities of transduced NPC and ESCC cells. We showed that NID2 significantly inhibits liver metastasis. Mechanistic studies of signaling pathways also confirm that NID2 suppresses the EGFR/Akt and integrin/FAK/PLCγ metastasis-related pathways. This study provides novel insights into the crucial tumor metastasis suppression roles of NID2 in cancers.

## INTRODUCTION

Cancer is a multifactorial disease arising as a consequence of both genetic and epigenetic alterations [1]. In recent decades, there has been intensified focus on the study of cancer epigenetics, with the aim to better understand the disease and develop novel therapeutics targeting epigenetic changes. Aberrant promoter hypermethylation of tumor suppressor genes (TSGs) and/or metastasis suppressor genes (MSGs) has been well-studied and is known to be a key driver of tumorigenesis in several cancers [2–4].

Nidogen-2 (NID2) encodes a secretory protein from the nidogen protein family [5]. The NID2 and also its family member, NID1, are ubiquitously present in the basement membrane and serve to maintain the integrity and stability of the basement membrane by connecting laminin and collagen IV networks in the extracellular matrix (ECM) [5, 6]. NID2 silencing was reported in many cancer types and the aberrant promoter hypermethylation is one of the most critical events detected in different malignancies, including gastric [7], bladder [8, 9], and invasive cervical [10] cancers. Detection of NID2 methylation was proposed as a biomarker for the diagnosis of many cancers including non-small cell carcinoma of lung [11], urothelial carcinoma of urinary bladder [9, 12, 13] and squamous cell carcinoma of oral cavity [14]. Furthermore, a functional study revealed that NID2-deficient mice have higher lung metastasis upon tail vein injection of melanoma cells [15], suggesting a critical role of NID2 in suppressing the metastatic potential of cancer cells.

Previously, we utilized the Illumina HumanMethylation450 Bead Chip (HM450) to examine the methylome profile of two cancers of the aerodigestive tract of relatively high prevalence in China, namely, nasopharyngeal carcinoma (NPC) [16] and esophageal squamous cell carcinoma (ESCC). A number of TSGs silenced by means of promoter hypermethylation has already been identified in these two cancers [17–23]. They are confirmed by our previous functional genetics and epigenetics approaches. From the methylome analyses, *NID2* was confirmed to be the top hit as a promoter hypermethylated gene in both NPC and ESCC. As *NID2* harbors several *de novo* methylated loci in the CpG islands, it is a potential TSG/MSG in these cancers.

Despite the substantial number of studies associating *NID2* to different cancers, to the best of our knowledge, there have not been any in-depth functional studies to elucidate the potential suppressive role of *NID2* in cancers, especially in NPC and ESCC. Hence, in the present study, we aimed to scrutinize the functional role of *NID2* in these cancers.

## RESULTS

### Down-regulation of NID2 is highly associated with aberrant promoter hypermethylation in both NPC and ESCC

**Figure 1:**
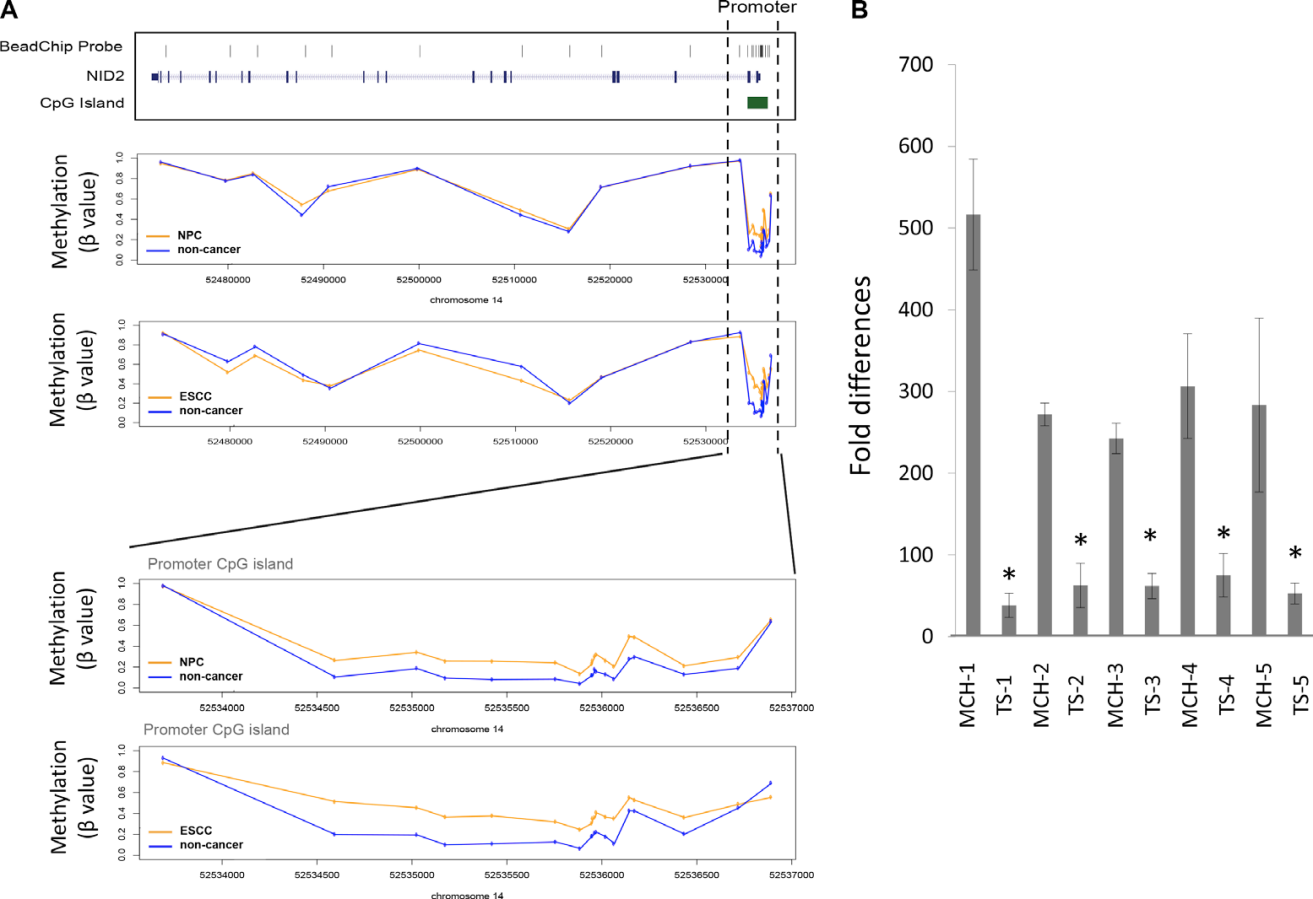
NID2 is identified as a candidate gene in NPC and ESCC (**A**) The average methylation level of *NID2* derived from our previous methylome data in NPC and ESCC. The vertical broken line shows the region covering the promoter CpG island (chr14: 52534582–52536722) and the bottom figures show a close-up view of changes in methylation. Methylation level is presented as β value (β = M/(U+M+100), M: signal intensity of the methylated allele, U: signal intensity of the unmethylated allele). The y-axis shows the average methylation level in tumors (orange line) and non-cancer controls (blue line), respectively. Within this region, the methylation levels in multiple CpG sites of both NPC and ESCC patients are consistently higher than those of non-cancer controls, with adjusted *p* value < 0.05 estimated by LIMMA analysis using the transformed β values as previously described [16]. Significance level of each selected probes were shown in Supplementary Figure S 1. (**B**) MMCT of chromosome 14 was previously performed using HONE1 as the recipient cell line [18]. qPCR analysis of tumor-suppressive microcell hybrids (MCHs) and their tumor segregant (TS) cell lines, which are no longer tumor-suppressive, showed that NID2 expression was down-regulated in all five TS cell lines, when compared to their respective MCHs. Asterisk (*) indicates samples with more than two-fold differences compared to its MCHs.

Our previous HM450 methylome analysis of 25 primary NPC [16] and 17 primary ESCC (unpublished data) and their matched adjacent non-cancer tissues has shown that hypermethylation is important in these cancers. This is a frequent event in NPC compared to many other cancer types [24]. In this study, we analyzed our NPC and ESCC methylome data to identify candidate genes that are regulated by aberrant methylation. Among the genes that showed differential methylation, *NID2* was one of the top candidate genes showing significant differences in the methylation levels between cancer and non-cancer specimens. In both NPC and ESCC, the CpG-rich promoter regions of *NID2* were hypermethylated, when compared to the matched non-cancer tissues (Figure 1A) (Supplementary Figure S1).

In addition to global HM450 methylome studies, our previous functional complementation studies utilized the microcell-mediated chromosome transfer (MMCT) approach to transfer an intact human chromosome 14 into the tumorigenic HONE1 cell line for identification of TSGs [18]. A panel of tumor-suppressive microcell hybrids (MCHs) and tumorigenic tumor segregants (TSs) was established. Studies found that TSGs usually show up-regulation in the MCHs, while being down-regulated in the TSs [18]. *NID2* showed significant up-regulation in the MCHs and down-regulation in the matched TSs (Figure 1B). This functional complementation study further supported the potential of the NID2 to function as a tumor suppressor or metastasis suppressor.

**Figure 2:**
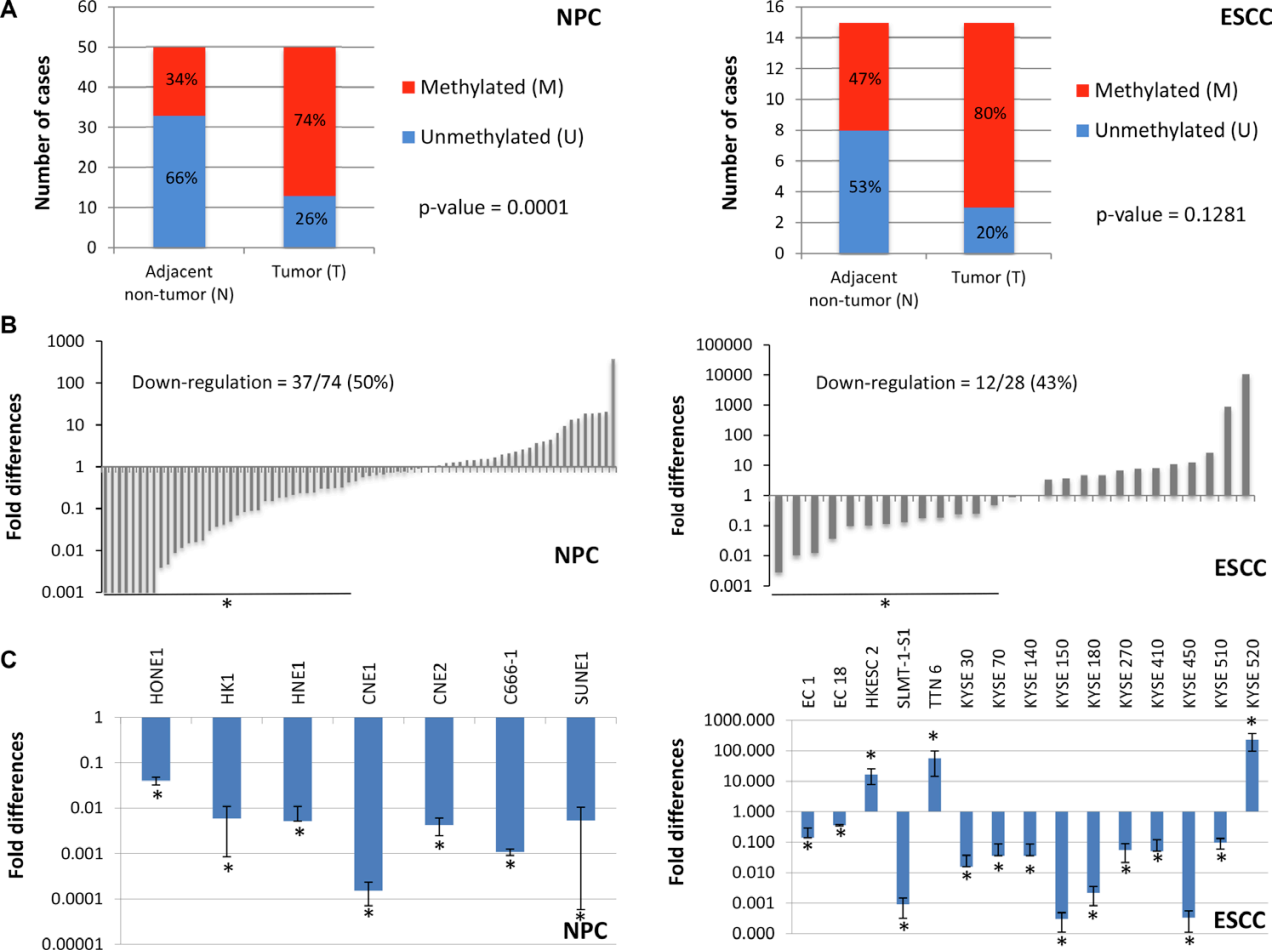
Validation of NID2 promoter hypermethylation and its down-regulation in NPC and ESCC (**A**) In 37 of 50 (74.0%, *p*-value = 0.0001) NPC biopsies, the *NID2* promoter hypermethylation was observed. The *NID2* promoter was methylated in 12 of 15 (80.0%, *p*-value = 0.1281) ESCC biopsies, as shown by MS-HRM results. (**B**) qPCR analysis showed that expression of NID2 is down-regulated in 50% (37/74) of NPC biopsies and 43% (12/28) of ESCC biopsies showing more than two-fold down-regulation of *NID2* expression. (**C**) All 7 NPC cell lines showed down-regulation of *NID2* expression, after normalization to the immortalized nasopharyngeal cell line. 75% of the ESCC cell lines (12 of 16) have decreased *NID2* expression, when compared to the immortalized normal esophageal epithelial cell line. The housekeeping gene *GAPDH* was used as an internal control for all qPCR analysis and the asterisks (*) indicate samples with more than two-fold up-regulation (> 2.0) or down-regulation (< 0.5).

To evaluate the *NID2* promoter hypermethylation in NPC and ESCC, we identified the region that showed distinct differential methylation in selected NPC cell lines, when compared to the NP cell lines (Supplementary Figure S2). The high-throughput methylation-sensitive high-resolution melting (MS-HRM) assay was used to assess the promoter methylation status of *NID2* in clinical samples. We found that 74% of 50 NPC biopsies had a methylated *NID2* promoter, while the majority of the adjacent non-cancer tissues remained unmethylated (66%) (Figure 2A). In ESCC, a trend of *NID2* promoter hypermethylation was detected in 80% of the samples (Figure 2A), which was consistent with our unpublished ESCC methylome data using the same samples. These MS-HRM results further validate the clinical significance of the *NID2* promoter hypermethylation, supporting our finding from the HM450. The MS-HRM results showed that the *NID2* promoter is methylated in HONE1, HK1, and C666 (NPC cell lines), when compared to the unmethylated immortalized nasopharyngeal cell lines (NP69 and NP460). Similarly, the *NID2* promoter is also methylated in the ESCC cell lines (KYSE30, KYSE150, and SLMT-1S1), while the *NID2* promoter in NE3 and NE083 (immortalized esophageal cell lines) remain unmethylated (Supplementary Figure S3).

The expression levels of NID2 were determined in paired NPC/ESCC and non-cancer biopsies to validate their clinical relevance. NPC [50% (37/74)] and ESCC biopsies [43% (12/28)] showed more than two-fold down-regulation of *NID2* (Figure 2B). These data support *NID2* being a potential TSG in ESCC and NPC. Immunohistochemical (IHC) analysis of NID2 was used to show protein expression levels directly in cancer tissues. Representative images are shown in Supplementary Figure S4, in which stronger staining in the non-neoplastic glands of nasopharyngeal mucosa was observed in comparison to the staining in NPC. qPCR analysis in the cell lines showed that *NID2* is down-regulated in 100% (7/7) of NPC cell lines and 80% (12/15) of ESCC cell lines (Figure 2C). Consistent with the MS-HRM results, *NID2* expression was lower in cell lines that had methylated *NID2* promoter (HONE1, HK1, C666-1, KYSE30, KYSE150, and SLMT-1S1), when compared to the respective non-neoplastic immortalized cell lines with an unmethylated *NID2* promoter.

### NID2 re-expression suppresses NPC and ESCC cancer-associated characteristics: Clonogenic survival and cancer cell migration and invasion abilities

**Figure 3:**
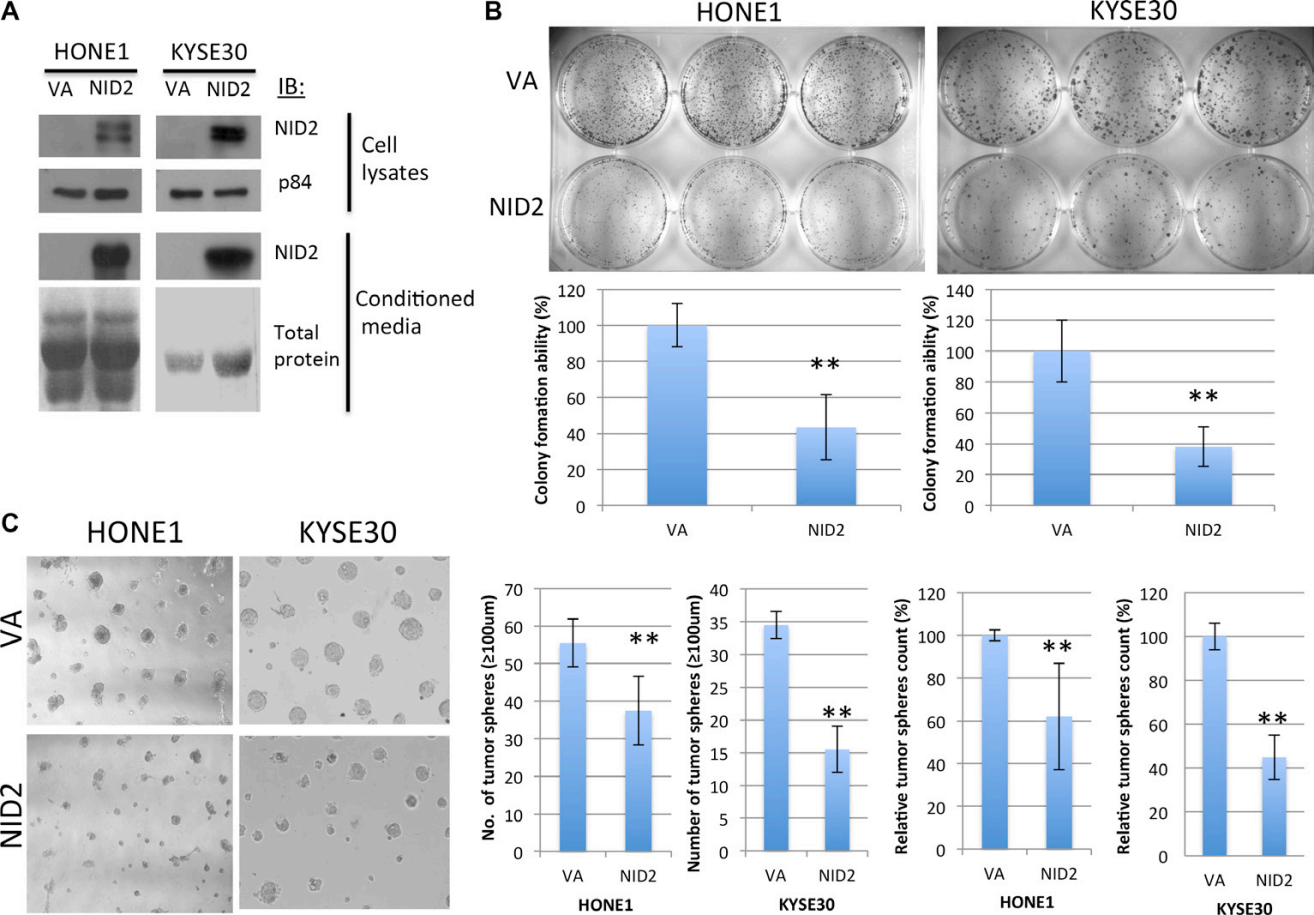
NID2 inhibits 2D and 3D clonogenic survival (**A**) Western blot analysis confirmed re-expression of NID2 in selected NPC (HONE1) and ESCC (KYSE30) cell lines, which have down-regulated NID2 expression. Over-expression of NID2 was detected in cell lysates, as well as being secreted into the conditioned media. Coomassie blue staining of total protein was used as a loading control for the conditioned media. (**B**) 2D colony formation assays of HONE1 and KYSE30 cells. Re-expression of NID2 significantly reduced their colony-forming abilities to around 40% (**p < 0.01). Data shown are the mean from three independent experiments ± S.D. (**C**) In 3D Matrigel culture, cells re-expressing NID2 resulted in significantly lower numbers of tumor spheres (≥ 100 μm), when compared to the VA in both NID2-expressing HONE1 and KYSE30 cells (**p < 0.01). Data shown are the mean from three independent experiments ± S.D.

To confirm the effect of NID2 on both NPC and ESCC disease progression, NID2 was re-expressed in NPC (HONE1) and ESCC (KYSE30) cells. Detectable NID2 protein was found in both cell lysates and conditioned media (Figure 3A). The NID2-expressing cells were then used for the downstream functional studies.

The clonogenic survival ability of tumor cells is a prerequisite for occurrence of metastasis and was assessed by 2D and 3D colony formation assays. NID2-expressing HONE1 cells significantly reduced colony-forming ability. The number of colonies formed was only 43.4% of the vector-alone (VA) control. KYSE30 cells expressing NID2 only retained 38% of the colony-forming ability (Figure 3B). The NID2-expressing cells were also cultured on the 3D Matrigel matrix, to mimic the ECM environment. They showed significant reduction of clonogenic survival ability. On average, the number of tumor spheres (≥ 100 μm) formed in NID2-expressing HONE1 and KYSE30 was reduced to 62% and 45%, respectively, of the VA control (100%) (Figure 3C).

**Figure 4:**
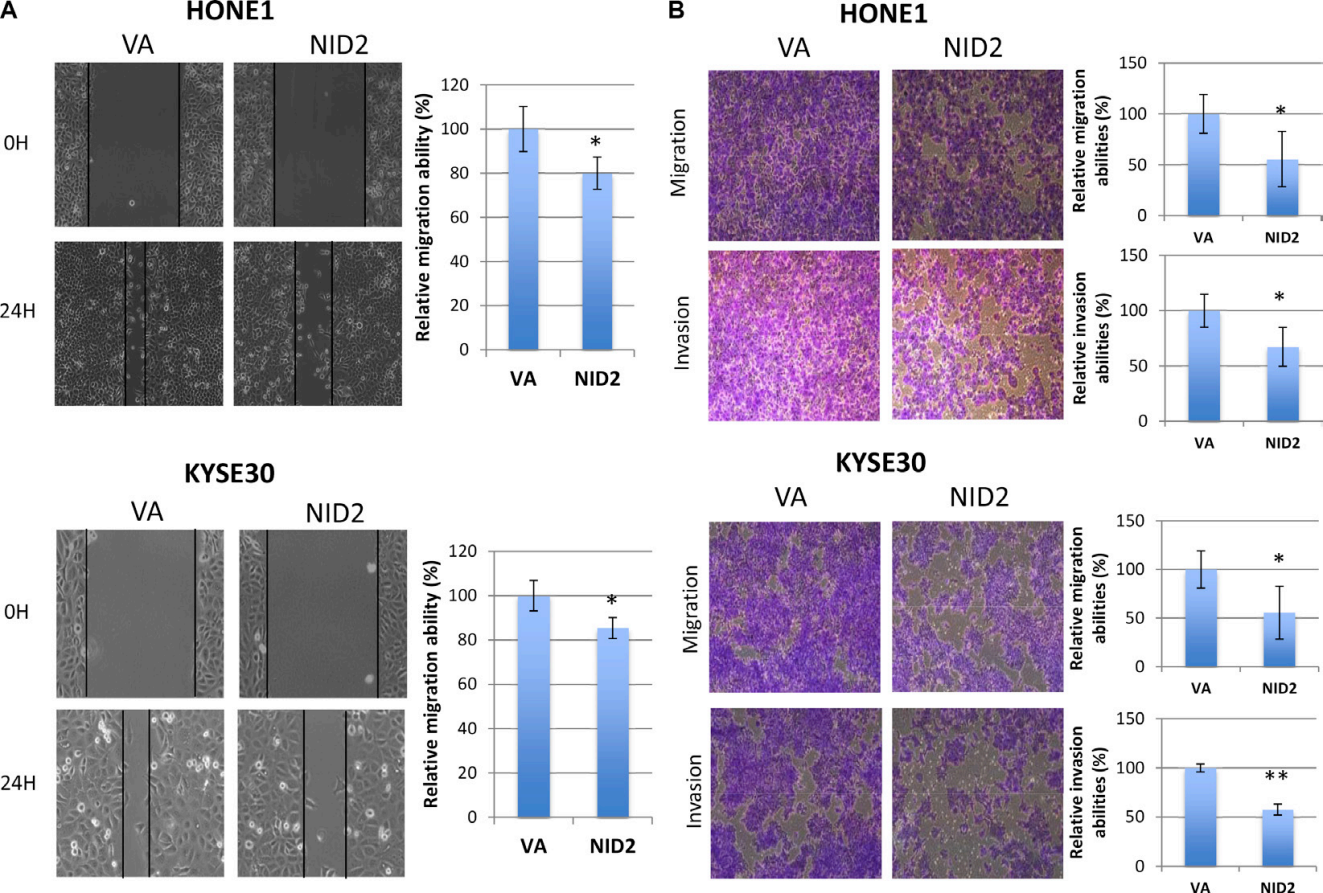
NID2 suppresses cell migration and invasion (**A**) Cell migration ability was evaluated by the wound healing assay. Microscopic images were taken at 0th hour and 24th hour to monitor the extent of wound closure. Both NID2-expressing HONE1 and KYSE30 cells showed impaired wound healing ability, when compared to the VA controls (*p < 0.05). (**B**) Representative fields of migrated or invaded cells at 100x magnification. Re-expression of NID2 resulted in significant reduction of migration and invasion abilities of HONE1 and KYSE30 cells (*p < 0.05, **p < 0.01). Data shown are the mean from three independent experiments ± S.D.

Previous study suggested that NID2-deficient mice have higher lung metastasis [15], implying a critical role of NID2 in suppressing cell motility and, thus, metastasis. We examined the ability of NID2 to inhibit *in vitro* migration in the wound healing assay. NID2 re-expression in both HONE1 and KYSE30 impaired migration ability, as seen by the delayed wound closure, when compared to VA control (Figure 4A). In the *in vitro* migration chamber assay, the relative migration abilities were also significantly lower in NID2-expressing HONE1 (68%, *p < 0.05) and KYSE30 (56%, *p < 0.05) (Figure 4B). In Matrigel-coated invasion chambers, re-expression of NID2 led to significant reduction in the relative invasiveness of HONE1 (67%, *p < 0.05) and KYSE30 (58%, **p < 0.01) (Figure 4B). These results confirmed the functional role of NID2 in suppression of *in vitro* cancer cell migration and invasion.

### *NID2 negatively regulates the cancer-related EGFR/Akt and Integrin/FAK/PLC*γ *pathways associated with ECM protein signaling*

**Figure 5:**
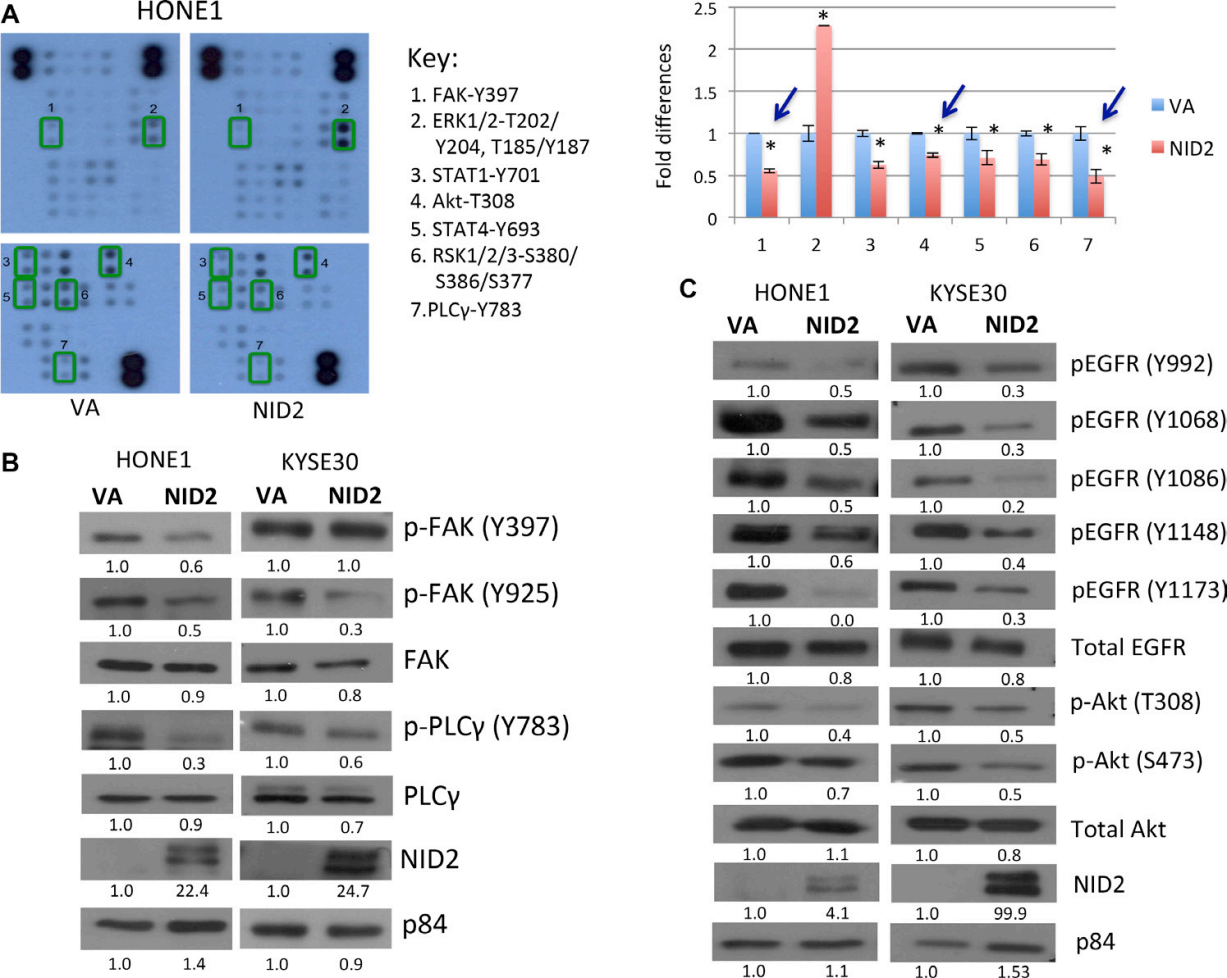
NID2 suppresses important signaling pathways in cancer (**A**) Phosphorylation study of NID2-expressing cells using the Human Phospho-Kinase Antibody Array. Reduced phosphorylation of (1) FAK (Y397), (3) STAT1 (Y701), (4) Akt (T308), (5) STAT4 (Y693), (6) RSK1/2/3 (S380/S386/S377), and (7) PLCγ (Y783), and increased phosphorylation of (2) ERK1/2 (T202/Y204, T185/Y187) in the NID2-expressing cells was observed. The bar chart shows the relative quantification of the array data, normalized to VA control (*p < 0.05). Blue arrows indicated significant changes that were further validated using Western blot. (**B**) Re-expression of NID2 in HONE1 and KYSE30 cell lines resulted in reduced phosphorylation levels of FAK (Y397/Y925) and PLCγ (Y783), as shown by representative Western blot images. (**C**) The levels of phosphorylated EGFR at five tyrosine sites (Y992/Y1068/Y1086/Y1148/Y1173) were decreased by the re-expression of NID2 in both HONE1 and KYSE30. Downstream Akt pathways were also suppressed by NID2, as shown by the reduced phosphorylation level of Akt (T308/S473). Numbers at the bottom of each band represent the relative quantification of the protein level, normalized to the VA control.

Studies of how NID2 may be involved in different cancer signaling pathways are still lacking. The Human Phospho-Kinase Antibody Array was utilized to explore the possible cancer signaling pathways that may be affected by NID2. NID2 re-expression significantly reduced the phosphorylation levels of several cancer-related molecules in the key ECM protein-related pathways, including FAK (Y397) and PLCγ (Y783) of the integrin signalling pathways and Akt (T308) of the epidermal growth factor receptor (EGFR) downstream signaling pathways (Figure 5A).

Cell-ECM interactions are crucial in regulating the behavior of cells. The signal transduction cascade induced by alteration in the ECM is mediated by integrins and EGFR. Members of the integrin-mediated ECM signaling pathways such as FAK and PLCγ have reduced phosphorylation upon NID2 expression, suggesting an overall inactivation of integrin signaling (Figure 5B). Western blot analyses on NID2-expressing cells validate our findings in the human phosphorylation array. Previous study suggested that the inhibition of EGFR/Akt signalling significantly suppresses cancer cell metastasis, including NPC metastasis [25]. NID2 re-expression in cancer cells revealed attenuated EGFR activity. There were reduced levels of the phosphorylated form of EGFR at various tyrosine sites (Y992, Y1068, Y1086, Y1148, and Y1173) (Figure 5C). NID2 also inhibited the downstream Akt pathway. Reduction in phospho-Akt levels was observed at T308 and Y473 sites (Figure 5C). These results support the crucial role for NID2 in the suppression of these cancer-associated signaling pathways.

### NID2 potently inhibits *in vivo* metastasis, but does not affect *in vivo* subcutaneous tumor growth

**Figure 6:**
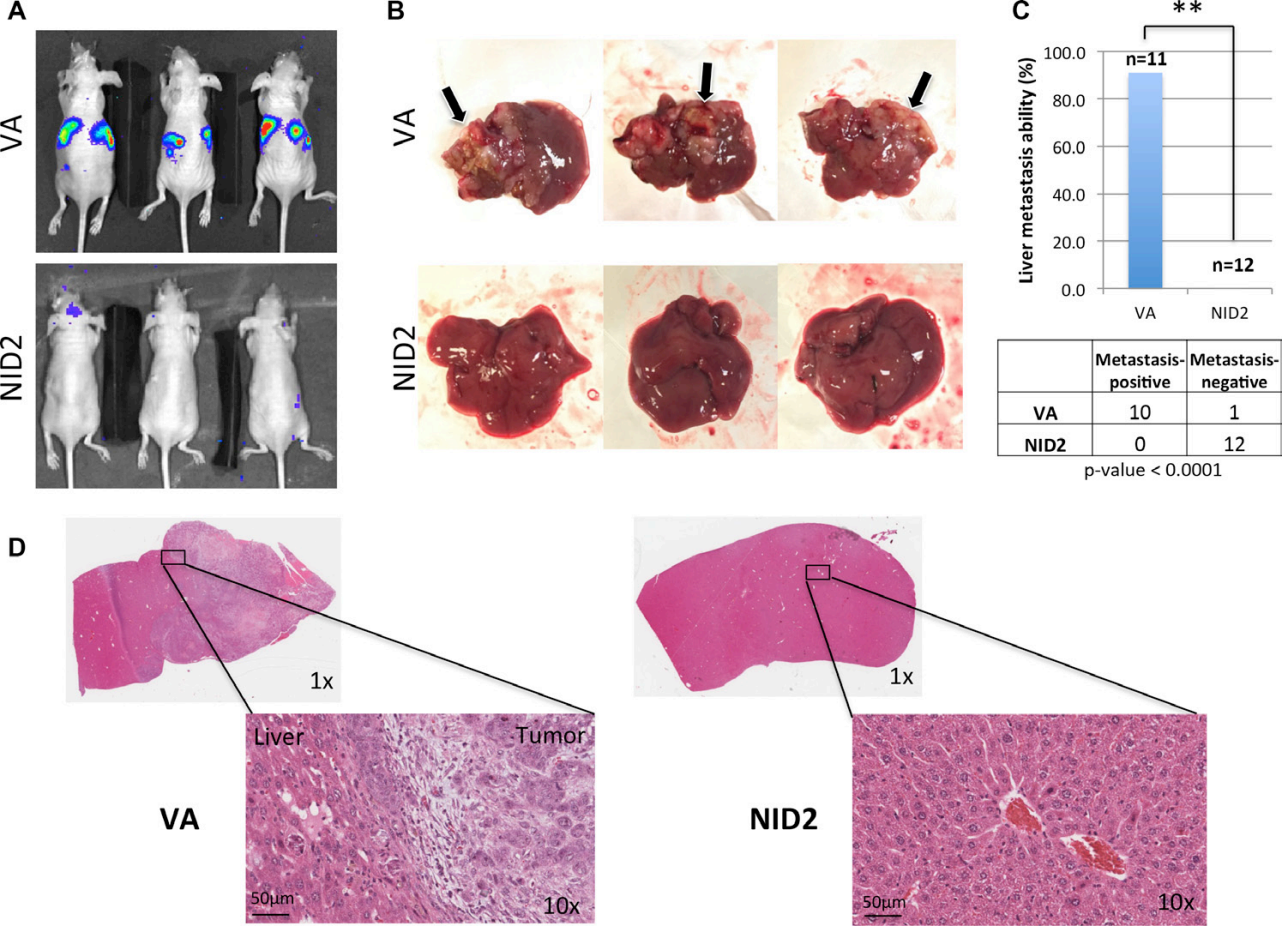
NID2 significantly suppresses cancer metastasis *in vivo* (**A**) Representative images of nude mouse bioluminescent imaging to monitor the extent of *in vivo* metastasis after intrasplenic injection (**B**) Lesions on liver were apparent to the naked eye, for those in which metastasis occurred, and are indicated with arrows. None of the excised livers in the NID2 group showed overt tumor lesions. (**C**) Comparison of *in vivo* metastasis ability between the two groups revealed a significantly strong suppression of liver metastasis in the NID2 group, in which 0 of 12 mice developed tumors, while in contrast, 10 of 11 mice in the vector control group developed liver metastasis (**p < 0.0001) (**D**) Histological examination of the excised livers confirms the presence of multiple tumors. Representative H&E stained images of mouse liver from the VA group with metastatic undifferentiated carcinoma and mouse liver from NID2-expressing group, with no observable tumor.

Previous study showed that NID2-deficient mice have a higher lung metastasis frequency, when intravenously injected with melanoma cells [15]. In this study, the functional role of NID2 in the suppression of *in vivo* metastasis was examined by intrasplenic injection of metastatic cancer cells. The NID2-expressing cells and the VA control were used for intrasplenic injection into nude mice [25] and the *in vivo* metastasis status was monitored weekly by the Xenogen live animal imaging system (Figure 6A). The nude mice were euthanized at week 3 post-injection, followed by necropsy. Using the highly metastatic HONE1-Luc cells, tumor nodules were clearly visible in 90% of the livers from the VA group (Figure 6B). The mice from the VA group showed a high metastasis rate to the liver (10 of 11 mice; 90%), whereas in the NID2 group, there were no metastasized tumors observed in the excised livers (0 of 12 mice; 0%; *p*-value = 0.0001) (Figure 6C). The results were further validated by H&E staining. Representative H&E staining images of metastatic liver tumor nodules and metastasis-negative samples are shown (Figure 6D). These results provide strong evidence for a functional role of NID2 in the inhibition of the *in vivo* metastasis.

**Figure 7:**
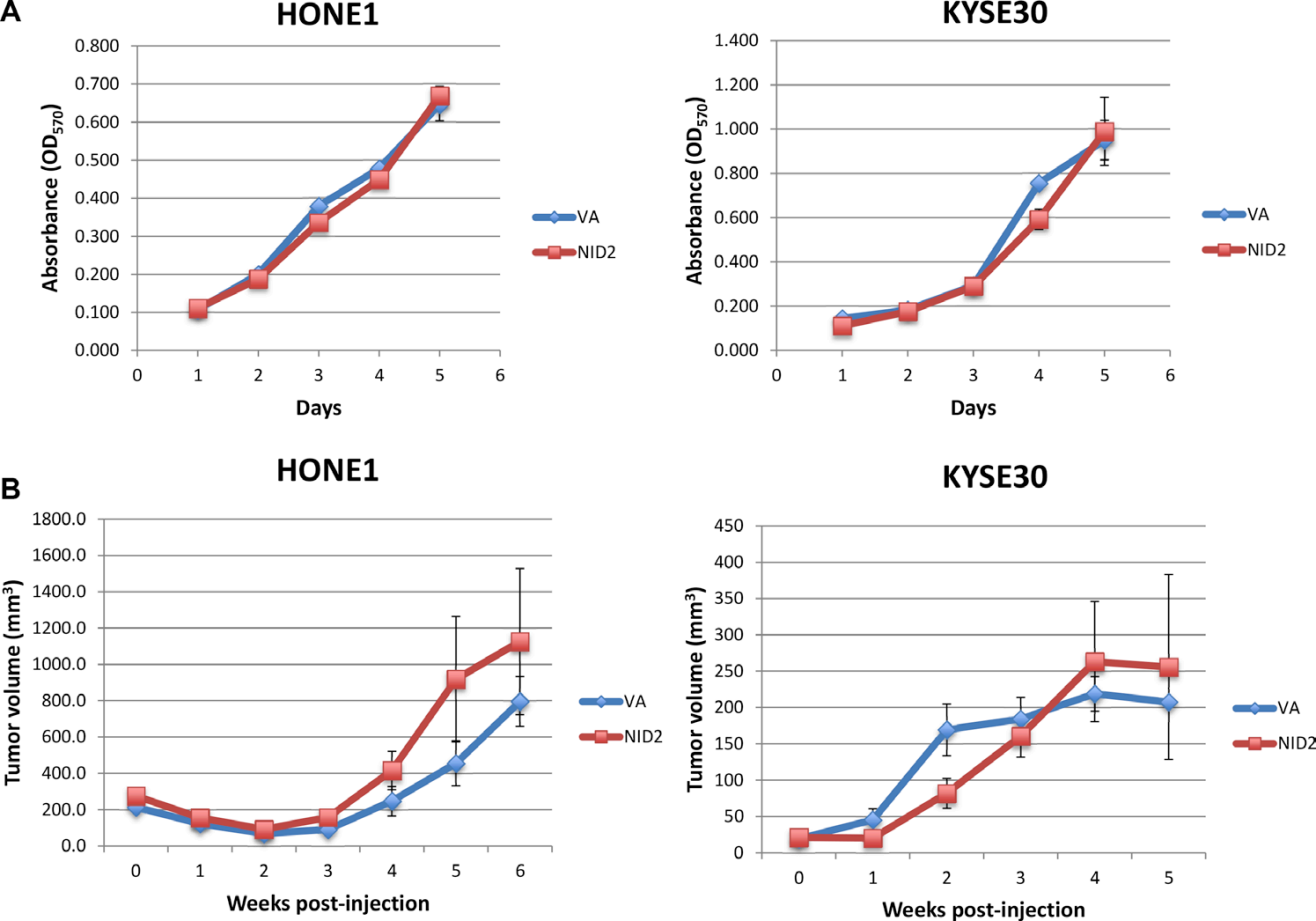
NID2 does not affect cell proliferation *in vitro* and tumor growth *in vivo* (**A**) *In vitro* cell proliferation was assessed using the MTT assay. Re-expression of NID2 did not affect HONE1 and KYSE30 cell growth. (**B**) Nude mouse subcutaneous injection was used to assess the effect of NID2 on *in vivo* tumor growth. Using both HONE1 and KYSE30, there were no significant differences in the tumor growth kinetics between the NID2-expressing and VA groups. Each data point was plotted as an average tumor volume of six tumor sites ± S.E.M.

In order to eliminate the possibility that the difference in the ability for metastasis is induced by the inhibition of the *in vivo* tumor growth by NID2, the *in vitro* cell proliferation assay and *in vivo* subcutaneous nude mouse tumorigenicity assay were performed. Results suggested that NID2 re-expression was unable to induce significant changes in both the *in vitro* cell proliferation and *in vivo* tumorigenicity of the cancer cells (Figure 7A and 7B). These results provide further evidence in support of NID2 playing an important role in metastasis suppression, despite it not affecting primary tumor growth.

## DISCUSSION

In this study, we examined the methylome of NPC and ESCC tumors and successfully identified a hypermethylated gene, *NID2,* which is a potential TSG/MSG. This possibility was further supported by our previous chromosome 14 MMCT NPC study. Frequent allelic deletions of chromosome 14q have also been observed in both NPC [26] and ESCC [27], where *NID2* gene is mapped. *NID2* was also consistently found to be hypermethylated in several other cancers [7–14, 28–30].

NID2 is an ECM protein and an important member of the basement membrane. Deregulated ECM signaling caused by a disrupted basement membrane network is widely recognized as a hallmark of cancer [31]. The dynamic and complex cell-matrix signal transduction orchestrates cell behavior, which functionally impacts its adhesion, migration, differentiation, and proliferation [32, 33]. Previously, we identified several TSGs in NPC and ESCC that are either encoding ECM proteins (*FBLN2* [21] and *LTBP2* [22, 34, 35]) or proteases involved in ECM remodelling (*ADAMTS9* [36, 37] and *MMP19* [38]*).* The re-expression of these TSGs was reported to suppress NPC and/or ESCC cell migration, invasiveness, angiogenesis, and *in vivo* tumor growth [21, 22, 34–36].

The colony formation assay is commonly used to assess the *in vitro* clonogenic survival ability, which is a critical prerequisite for cancer cells to colonize a secondary site during metastasis [39, 40]. In this study, concordance with the hypothesis that NID2 is a MSG, NID2 re-expression significantly suppresses the colony formation of both NPC and ESCC cells, in both 2D and 3D Matrigel culture. Furthermore, NID2 also plays a significant role in impairing the migration and invasion abilities of the cancer cells. These *in vitro* findings support our subsequent *in vivo* results. NID2-expressing NPC cells significantly inhibit metastasis formation in the liver. Collectively, these results confirm the role of NID2 as a potent suppressor of metastasis in NPC.

Metastasis is a complex process that involves dynamic degradation and remodelling of the ECM. There is increasing recognition of the important role of the ECM-defined tumor microenvironment in tumor initiation and progression. This is exemplified by the discovery of how ECM collagen regulates ECM stiffness and triggers a signaling network to promote breast cancer cell invasion and metastasis [41, 42]. Similarly, Mokkapati et al. reported higher lung metastases for melanoma cells in NID2-null mice [15]. The absence of this important component of the vascular basement membrane facilitated the transmigration of melanoma cells and resulted in a higher metastasis frequency to the lung [15]. This is consistent with findings in this current study, where the re-expression of NID2 significantly inhibited NPC cell metastasis to the liver. The abilities of tumor cells to survive in the blood circulation, to extravasate and to colonize the distant metastatic organ are the key steps governing metastasis, which can all be evaluated in our *in vivo* metastasis assay. In our study, NID2-expressing cells have markedly lost these abilities and, as a result, none of the mice in the NID2 group had distant metastasis. In contrast, the VA group had a very high percentage of distant metastasis (90%), with obvious tumor nodules on the liver. We hypothesize that the tumor microenvironment of NID2-expressing cells in the circulation or liver disfavors the processes of extravasation and colonization during the later cascade of metastasis, leading to the observation of distinctive difference in metastatic outcome, when compared to the control cells. Thus, the down-regulation of *NID2* by promoter hypermethylation appears to be a key event leading to cancer metastasis.

As a secretory basement membrane protein constituting the tumor microenvironment, changes in NID2 protein levels can lead to a change in the ECM signaling cascade to redefine cell fate. Integrin molecules are the major receptors mediating extracellular signal transduction into regulating cellular responses such as migration and proliferation [32, 43–45]. It is conventionally believed that upon ECM ligand activation, integrin clusters, and signaling complexes are formed at the focal adhesions, including FAK, Src, PI3K, and PLCγ [46, 47]. Among these, activation of the FAK pathway is known to confer a survival advantage and promote cell motility [43, 47]. In our study, results from both the Human Phospho-Kinase Antibody Array and Western blot show reduction of phosphorylation in integrin-related downstream molecules such as FAK and PLCγ, when NID2 is re-expressed. The EGFR/PI3K/Akt pathways are important players in both NPC [25, 48] and ESCC [49, 50]. Previous study suggested that inhibition of the Akt signalling pathway greatly inhibits *in vivo* metastasis [25]. Overexpression of EGFR and/or up-regulation of EGFR phosphorylation signaling are common in both cancers [25, 48–50], with Akt playing a central role as the downstream effector molecule [48]. Interestingly, EGFR signaling pathways can also be activated by crosstalk with the integrin pathways, in which they may act synergistically to promote tumorigenesis and metastasis [51, 52].

In this current study, we found that restoration of NID2 expression has a negative regulatory role on both EGFR and integrin signaling pathways. We propose that NID2 elicits its *in vitro* migration/invasion suppression and *in vivo* metastasis inhibition effects via the negative modulation of these two oncogenic pathways. Further in-depth mechanistic study of how NID2 intervenes with these signaling networks is warranted to further elucidate our understanding of its ability to suppress metastasis. In conclusion, NID2 plays an important role as an ECM protein defining the tumor microenvironment and is involved in metastasis suppression.

## MATERIALS AND METHODS

### Cell culture

All NPC cell lines (HONE1, HK1, CNE1, CNE2, HNE1, SUNE1, and C666) and the immortalized nasopharyngeal epithelial cell lines (NP460 and NP69) were cultured as previously described [53–57]. The HONE1-Luc used for the intrasplenic injection was cultured as previously demonstrated [25]. The panel of tumor-suppressive microcell hybrids (MCHs) and tumorigenic tumor segregants (TSs) was established in an earlier chromosome 14 microcell-mediated chromosome transfer study and cultured as previously described [18]. The ESCC cell lines (KYSE30/70/140/150/180/270/410/450/510/520, SLMT-1S1, EC1, EC18, HKESC2, and T.Tn6) and the immortalized esophageal epithelial cell lines (NE3 and NE083) were cultured, as detailed [58, 59]. The 293FT cell line was purchased from Invitrogen (Carlsbad, CA, USA) and cultured as recommended. All cell lines were tested for mycoplasma.

### Clinical samples

For methylation-sensitive high-resolution melting (MS-HRM) analysis, 50 NPC biopsies and matched adjacent non-cancer tissues were collected in Hong Kong between 2010–2013. These specimens were a subset of our previous methylation biomarker study [60]. A total of 17 archived paired ESCC biopsies were obtained from Tuen Mun Hospital and Queen Mary Hospital for the ESCC methylome study (unpublished) and 15 of the informative cases were used in MS-HRM. Specimens utilized for the qPCR were as previously described [18, 59].

### Extraction and bisulfite conversion of genomic DNA

Genomic DNA from NPC and ESCC biopsies was extracted using TRIzol (Invitrogen, Carlsbad, CA, USA), as described previously [60]. Genomic DNA was extracted from cell lines using a previously published phenol-chloroform extraction protocol [61]. A quantity of 500 ng genomic DNA was used as input for bisulfite conversion [62]. Bisulfite-converted DNA was then precipitated with ethanol and resuspended in 50 μl of 10 mM Tris-HCl (pH 8.5).

### Identification of putative NID2 promoter

Sequences within 200 base pairs of the transcription start site (TSS200) and 5′ untranslated region (5′UTR) sequences in the first exon of NID2 were retrieved from the UCSC browser (GRCh37: chr14: 52535485-52536146). Putative promoter sequences were identified using PROSCAN (http://www-bimas.cit.nih.gov/molbio/proscan/) (Supplementary Figure S5). Using MethPrimer [63], a CpG island of 324bp was identified and a pair of primers was designed to amplify a 293bp region within this island for clonal bisulfite sequencing. The primers used were 5′- TAGTTTGTTGGGTGGGTTTG -3′ and 5′- CCTTCCTACAAAAACTAATCCCC-3′ (Supplementary Figure S6).

### Methylation-sensitive high-resolution melting (MS-HRM)

Primers for MS-HRM were designed based on guidelines from Wodjaz et al. (2008) [64]. An 88bp region containing 12 CpG sites was amplified using the following MS-HRM primers: 5′- CGCGGAGAGTGGGTTGGAGGT -3′ and 5′- ACCACCCGATCCCCCTCCATACT -3′. MS-HRM analysis was carried out using the Light Cycler 480 System (Roche), as described [60].

### Immunohistochemical (IHC) analysis

IHC staining was performed on NPC biopsies using anti-NID2 (SAB1409003) polyclonal antibody from Sigma-Aldrich (St Louis, MO, USA), following procedures as previously reported [65].

### Plasmid construction and lentiviral infection

The full-length human *NID2* gene, *in* pCEP-Pu-NID2 vector, was provided by Dr. T Sasaki (Friedrich-Alexander-University, Germany). The NID2 open reading frame (ORF) was cloned into a lentiviral expression vector, pLenti6/UbC/V5-DEST (Invitrogen, Carlsbad, CA, USA). The vector-alone (VA) and *NID2* constructs were co-transfected with the packaging vector, psPAX2 and the envelope vector, pMD2.G, at a ratio of 4:3:1 (8μg/6μg/2μg). The human embryonic kidney cell line 293FT was used for virus packaging. Viruses collected 72 hours post-transfection were filtered and stored at −80°C until use. For *in vivo* study, the *NID2* ORF was cloned into the pLVX-EF1α system and stably transduced cell lines were established.

### Quantitative RT-PCR analysis

Total RNAs were extracted using TRIzol (Invitrogen, Carlsbad, CA, USA). The cDNA was synthesized using M-MLV Reverse Transcriptase (USB, Cleveland, OH, USA) with 1μg of total RNAs. Expression levels of *NID2* were measured using quantitative RT-PCR with the Applied Biosystems Step-One Plus platform. NID2 and GAPDH Taqman probes were used. Up-regulation is defined as having a fold difference > 2.0, while down-regulation is defined as having a fold difference < 0.5.

### Western blot

Cells were grown to 60–80% confluence and conditioned media/lysates were harvested. Conditioned media was spun down at 4°C using 10K Amicon centrifugal filter (Millipore, Bedford, MA, USA) to concentrate the secreted protein. Cells were lysed using RIPA buffer and Western blot analyses were performed as previously described [17]. Antibodies used in this study are summarized in Supplementary Table S1. Quantification of the bands was performed using ImageJ software (National Institutes of Health, Bethesda, MD USA) [66].

### Colony formation assay

The 2D and 3D colony formation assays were performed as described by Kan et al. [35] and Wong et al. [67], respectively. For 3D colony formation assay, the 12-well plate was coated with one layer of Matrigel (BD Biosciences, San Jose, CA, USA). Transduced cells (2 × 10^4^) were seeded onto the Matrigel and allowed to grow for a week until tumor spheres became apparent. Ten images of different microscopic views at 100× magnification were captured and tumor spheres with diameters above 100 μm were counted.

### Wound healing assay

The wound healing assay was carried out as reported previously [65]. Wound closure percentage was calculated using the formula: (migrated distance at 24 hour ÷ initial wound width) × 100. For relative migration ability, the percentage wound closure of NID2-expressing cells was normalized to that of vector control.

### Transwell migration and invasion assays

The transwell assays were performed as described previously [35, 56]. Briefly, 2 × 10^5^ cells were seeded in serum-free medium in the top chamber of the BD BioCoat migration insert or Matrigel invasion insert (BD Biosciences, St Jose, CA, USA), fitted with an 8μm pore membrane. Culture medium enriched with 10% serum was used as chemoattractant in the bottom chamber. At the end of assay (24–48 hours), migrated and invaded cells were fixed and stained with crystal violet. Five different microscopic fields (100× magnification) of the chambers were taken and quantification was with ImageJ software (NIH, Bethesda, MD USA).

### Human phospho-kinase antibody array

The protein lysates from the vector-alone (VA) and NID2-expressing HONE1 cells were used for the Human Phospho-Kinase Antibody Array (R&D Systems, Minneapolis, MN, USA), as described [25]. Quantification of the array data was performed using ImageJ software (NIH, Bethesda, MD USA) [66].

### Cell proliferation assay

MTT (3-[4,5-dimethylthiazol-2-yl]-2,5 diphenyl tetrazolium bromide) assay was utilized for the *in vitro* cell proliferation assay as previously described [56]. A total of 2 × 10^4^ cells was seeded in 96-well plates and absorbance of MTT was measured daily for five consecutive days at 570 nm.

### *In vivo* tumorigenicity and metastasis assays

*In vivo* nude mouse subcutaneous injection was performed as described previously [57]. Briefly, 1 × 10^7^ and 1 × 10^6^ cells of transduced HONE1 and KYSE30 cells, respectively, were inoculated subcutaneously into the flanks of female BALB/cAnN-nu (nude) mice. Tumor growth was monitored weekly with the use of digital calipers.

Nude mouse intrasplenic injection was conducted for the *in vivo* metastasis assay as described [25]. A total of 1 × 10^6^ cells of pLVX-EF1α (VA)/pLVX-NID2 transduced HONE1-Luc was injected into the spleen of the mice following laparotomy. Mice were monitored weekly by detecting the bioluminescent signals upon intraperitoneal injection of D-luciferin as described [25]. Live bioluminescent imaging was performed using the IVIS Spectrum *In vivo* Imaging System (PerkinElmer, Norwalk, CT, USA), available at the Core Facility of Li Ka Shing Faculty of Medicine, The University of Hong Kong. At the third week post-inoculation, mice were sacrificed and necropsy was performed. The livers of the mice were excised and examined for presence of metastasized tumors. All the animal experiments were conducted with valid license from the Department of Health, Hong Kong S.A.R. and approved by the Committee on the Use of Live Animals in Teaching and Research (CULATR) of The University of Hong Kong.

### Paraffin embedding and Hematoxylin and Eosin (H&E) staining

Excised tissues were paraffin embedded and H&E stained as described [25].

### Statistical analysis

Chi-square test or Fisher's exact test (if any cell size was less than 5) was used in the MS-HRM analysis of biopsies and the *in vivo* metastasis assay. Student's *t*-test was utilized for statistical analyses for other experiments unless stated otherwise. *P*-values below 0.05 were considered statistically significant.

## SUPPLEMENTARY MATERIALS


