# Supplementary material for: Metastasis-suppressing NID2, an epigenetically-silenced gene, in the pathogenesis of nasopharyngeal carcinoma and esophageal squamous cell carcinoma
Source: Oncotarget. 2016 Oct 25;7(48):78859–71. doi: 10.18632/oncotarget.12889 (PMC5346683; doi:10.18632/oncotarget.12889)
Supplement: Supplementary file 1 [file oncotarget-07-78859-s001.pdf]

Metastasis-suppressing *NID2*, an epigenetically-silenced gene, in the pathogenesis of nasopharyngeal carcinoma and esophageal squamous cell carcinoma

Supplementary Materials

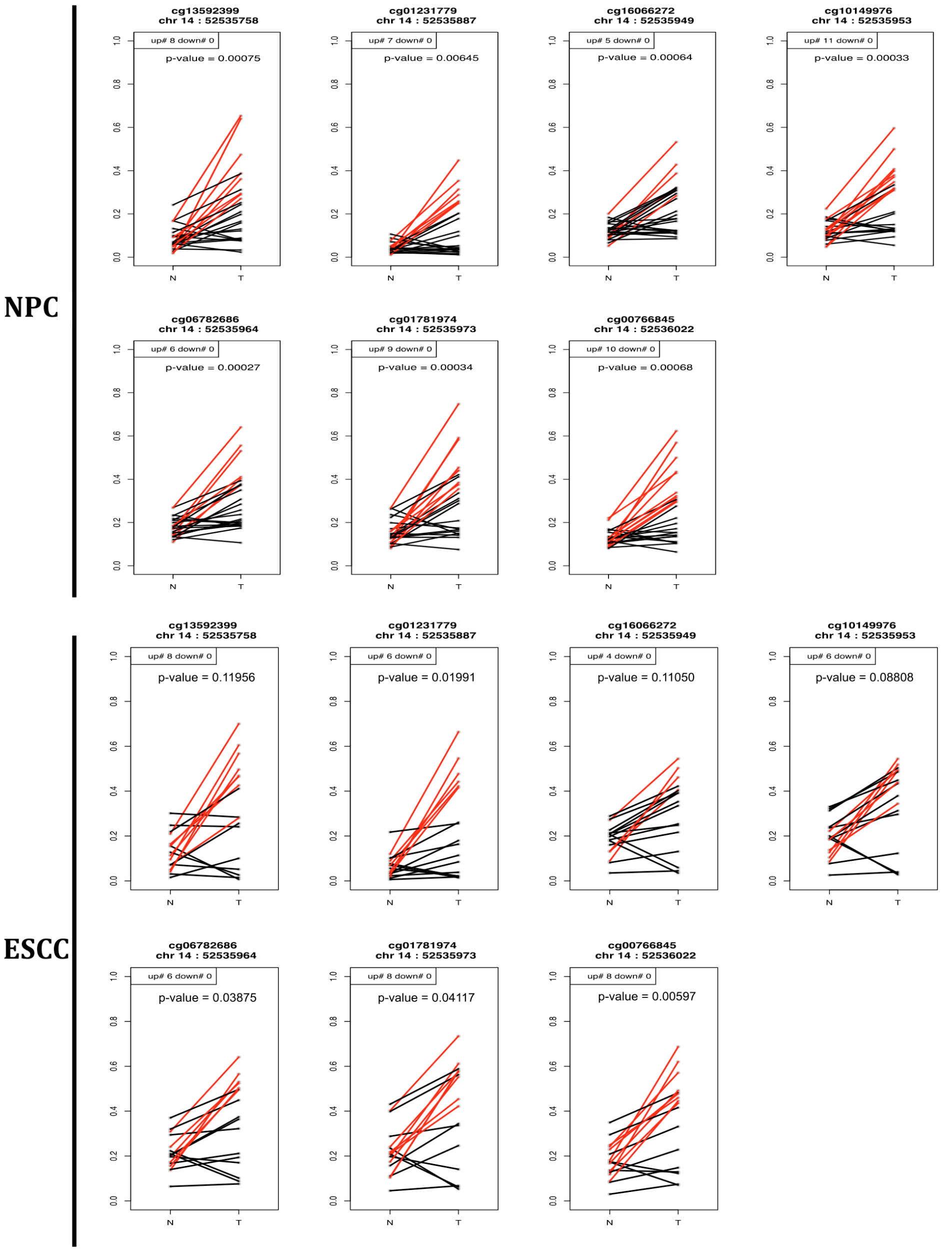

Supplementary Figure S1: Methylation level measured by HM450 at the selected probes located within the NID2 promoter region in both NPC (n = 25) and ESCC (n = 17). The y-axis shows the average methylation level, presented as β value. There is a general trend of increased methylation level in T (tumor – NPC/ESCC), relative to controls, N (non-tumor). Up = hypermethylated; Down = hypomethylated. $|\Delta\beta| > 0.2$ was considered as differential methylation and the *p*-values were indicated for each probe.

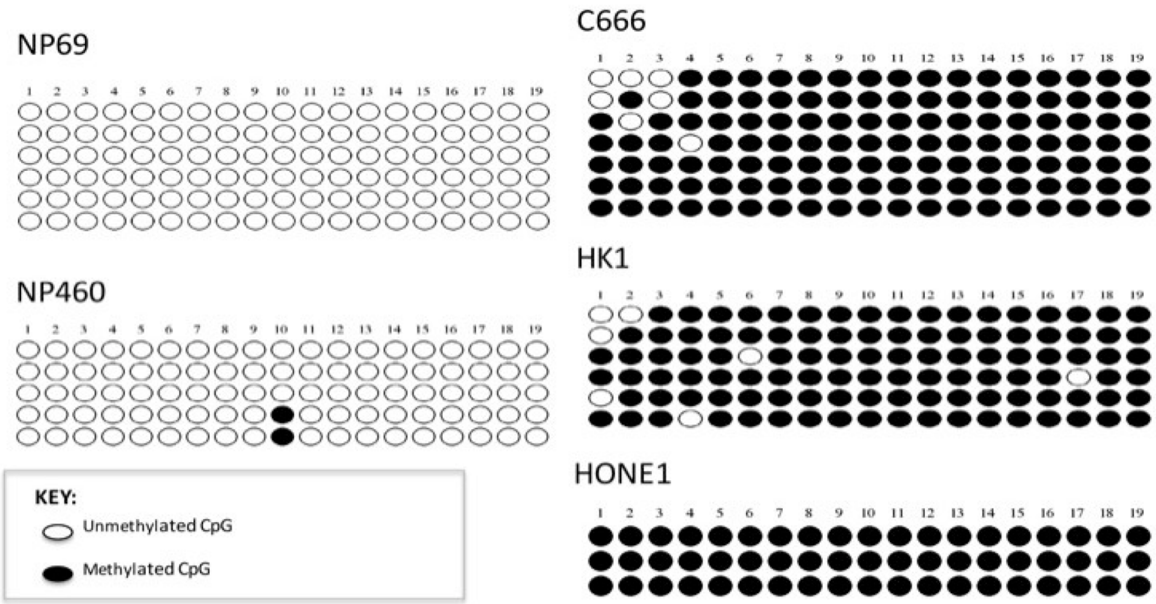

Supplementary Figure S2: Using bisulfite-treated genomic DNA of selected NPC cell lines as template, the CpG-rich promoter region was amplified using the primer designed by MethPrimer. The purified PCR products were cloned into the TA cloning vector, pMD18T (TakaRa Biotechnology, Dalian, China) and used to transform DH5α competent cells. Sequencing results were analyzed by the QUMA program (http://quma.cdb.riken.jp/) from RIKEN Center of Developmental Biology. Results shown that most CpG sites in NPC cell lines were methylated, while the CpG sites were mostly unmethylated in the two immortalized nasopharyngeal epithelial cell lines, NP69 and NP460.

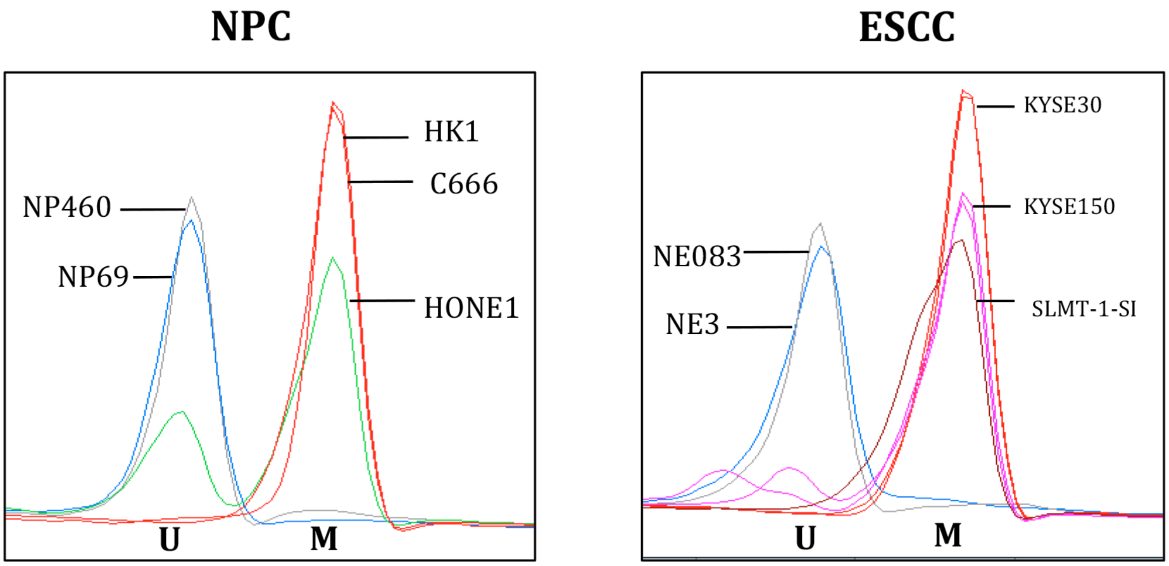

Supplementary Figure S3: MS-HRM analysis of NID2 promoter methylation in NPC and ESCC cell lines for pre-screening. All NPC and ESCC cell lines tested have a methylated peak (M), while the normal immortalized epithelial cell lines are unmethylated (U).

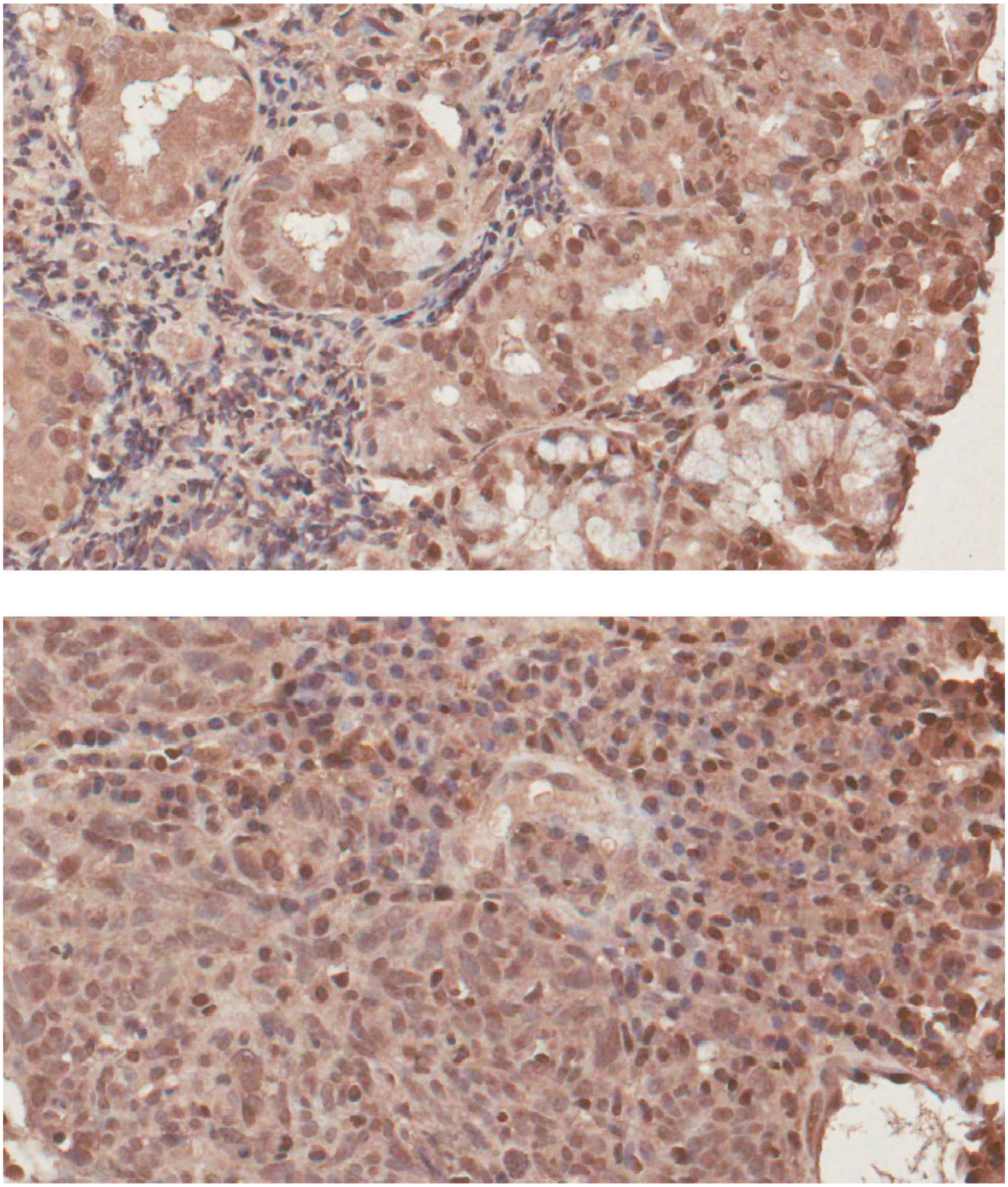

Supplementary Figure S4: Immunohistochemical (IHC) staining of NID2 in adjacent non-neoplastic nasopharyngeal mucosa (top) and NPC (bottom). Stronger staining was observed in the nuclei of the non-neoplastic glands of nasopharyngeal mucosa, while weaker and variable NID2 staining was observed in the nuclei of NPC. Images are 20× (top) and 25× (bottom) magnifications.

Lower case – 200bp upstream of Transcription Start Site (TSS200) | Position relative to TSS

Upper case – First exon sequence

Underlined <u>ATG</u> – translation start site

```
<
ctgagctcatctcctgcaacgccgctgccccaaaccttgcgggccatttg  -200
gtcccgggggtggggctccttgggggccgcggggcagcccggatgagagg  -150
atgacccgggagctccctgggggcggagtggaaggggcagctccaggcag  -100
ccctggccaggagcttttaccccgccagccccgctgctccagcggccgct  -50
GCCTTAGAAAAGTTAACGAGAACCAGATGTGGTGGCCACTGCCGAACTTT  50
CTCAGAGCCGGTGATTGGTCCCCAGCCGAGGGCCTCAGCCAATTAGCTTG  100
CTGGGTGGGCCTGGAGTCCCGCCCCGCCCAGGCGCCCGCGGAGATCCAGG  150
TTCGAGGCTGGCGCGGCGCGGAGAGTGGGCTGGAGGCCGGGGCGGGACGC  200
GTTGTGCAGCGGGTAAGCGCACGGCCGAGCGAGCATGGAGGGGGACCGGG  250
TGGCCGGGCGGCCGGTGCTGTCGTCGTTACCAGTGCTACTGCTGCTGCCG  300
TTGCTAATGTTGCGGGCCGCGGCGCTGCACCCAGACGAGCTCTTCCCACA  350
CGGGGAGTCGTGGGGGGACCAGCTCCTGCAGGAAGGCGACGACGAAAGCT  400
CAGCCGTGGTGAAGCTGGCGAATCCCCTGCACTTCTACGAAGCCCGATTC  450
AGCAACCTCTAC
>
```

Supplementary Figure S5: TSS 200 and the first exon (including 5′UTR) of NID2 gene on reverse strand of chromosome 14: 52535485-52536146 (662bp) were retrieved from the UCSC Genome Browser. PROSCAN Version 1.7, developed by Dr. Dan Prestridge (Advanced Biosciences Computing Center, University of Minnestota) was used to predict the promoter sequences from the above region. (http://www-bimas.cit.nih.gov/cgi-bin/molbio/proscan) Sequences of 251bp highlighted in grey were the putative promoter sequences of NID2, which encompass the TSS200 and 5'UTR region of the first exon. (chr14: 52535751-52536001)

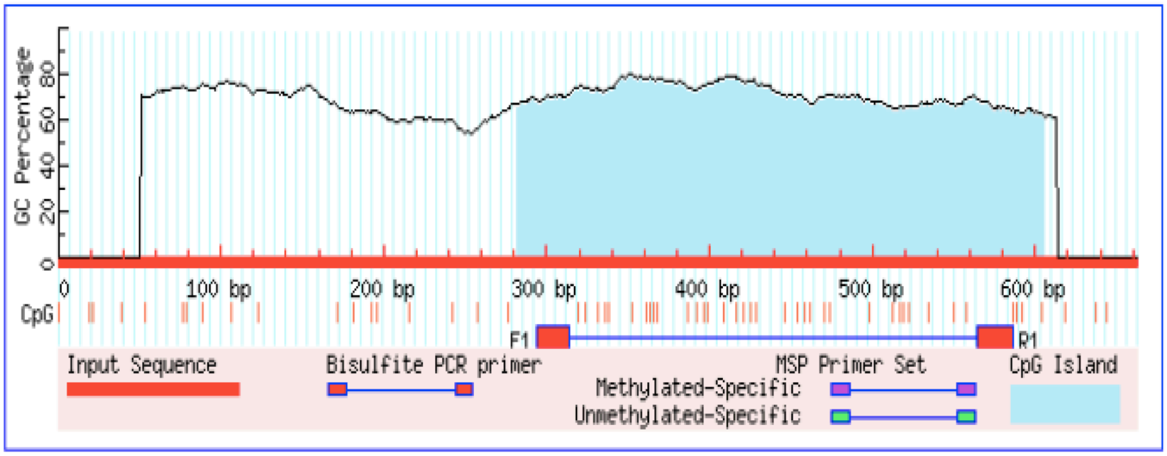

Sequence Name:
Sequence Length: 662

CpG island prediction results
(Criteria used: Island size > 100, GC Percent > 50.0, Obs/Exp > 0.6)
1 CpG island(s) were found in your sequence

	Size	(Start - End)
Island 1	324 bp	(282 - 605)

Primer picking results for bisulfite sequencing (or restriction) PCR

	Primer	Start	Size	Tm	GC%	'C's	Sequence
1	Left primer	294	20	58.38	65.00	4	TAGTTTGTTGGGTGGGTTTG
	Right primer	586	23	59.31	69.57	6	CCTTCCTACAAAAACTAATCCCC

Product size: 293, Tm: 74.2, CpGs in product: 33

Supplementary Figure S6: MethPrimer was used to predict the CpG island present in the TSS200/5'UTR region of NID2 and to design the primers for clonal bisulfite sequencing.

Supplementary Table S1: Summary of primary antibodies used in this study

Primary antibody	Host	Dilution	Size (kDa)	Source	Catalogue number
NID2	Goat	1:1000	200	R & D Systems	AF3385
p84	Mouse	1:1000	84	Genetex	GTX70220
pEGFR (Y992)	Rabbit	1:1000	170	Genetex	GTX25638
pEGFR (Y1068)	Rabbit	1:1000	170	Genetex	GTX61057
pEGFR (Y1086)	Rabbit	1:1000	170	Genetex	GTX61058
pEGFR (Y1148)	Rabbit	1:1000	170	Genetex	GTX10643
pEGFR (Y1173)	Rabbit	1:1000	170	Genetex	GTX61052
EGFR	Rabbit	1:2000	170	Cell Signaling	#4267
pAKT (T308)	Rabbit	1:1000	60	Cell Signaling	#4052
pAKT (S473)	Rabbit	1:1000	60	Cell Signaling	#4058
AKT	Rabbit	1:2000	60	Cell Signaling	#9272
pFAK (Y397)	Rabbit	1:1000	125	Cell Signaling	#3283
pFAK (Y925)	Rabbit	1:1000	125	Cell Signaling	#3284
FAK	Rabbit	1:1000	125	Cell Signaling	#3285
